# Immuno-pathomechanism of liver fibrosis: targeting chemokine CCL2-mediated HIV:HCV nexus

**DOI:** 10.1186/s12967-014-0341-8

**Published:** 2014-12-10

**Authors:** AW Wahid Ansari, Reinhold E Schmidt, Esaki M Shankar, Adeeba Kamarulzaman

**Affiliations:** Centre of Excellence for Research in AIDS, Faculty of Medicine, University of Malaya, Lambah Pantai, 50603 Kuala Lumpur Malaysia; Department of Medicine, Faculty of Medicine, University of Malaya, Lambah Pantai, 50603 Kuala Lumpur Malaysia; Department of Clinical Immunology and Rheumatology, Hannover Medical School, Carl-Neuberger Str.1, D-30625 Hannover, Germany; Department of Medical Microbiology, Faculty of Medicine, University of Malaya, Lambah Pantai, 50603 Kuala Lumpur Malaysia; Tropical Infectious Diseases Research and Education Centre, Faculty of Medicine, University of Malaya, Lambah Pantai, 50603 Kuala Lumpur Malaysia

**Keywords:** HIV/Hepatitis-C co-infection, Immuno-pathogenesis, Liver fibrosis, C-C chemokine ligand-2, Inflammation, Combination anti-retroviral therapy, Hepatic stellate cells, Viral cross-talk

## Abstract

Even in the era of successful combination antiretroviral therapy (cART), co-infection of Hepatitis C virus (HCV) remains one of the leading causes of non-AIDS-related mortality and morbidity among HIV-positive individuals as a consequence of accelerated liver fibrosis and end-stage liver disease (ESLD). The perturbed liver microenvironment and induction of host pro-inflammatory mediators in response to HIV and HCV infections, play a pivotal role in orchestrating the disease pathogenesis and clinical outcomes. How these viruses communicate each other via chemokine CCL2 and exploit the liver specific cellular environment to exacerbate liver fibrosis in HIV/HCV co-infection setting is a topic of intense discussion. Herein, we provide recent views and insights on potential mechanisms of CCL2 mediated immuno-pathogenesis, and HIV-HCV cross-talk in driving liver inflammation. We believe CCL2 may potentially serve an attractive target of anti-fibrotic intervention against HIV/HCV co-infection associated co-morbidities.

## Introduction

Liver fibrosis is an immuno-pathological event associated with chronic inflammation caused by liver injury and/or infection by the viruses. Uncontrolled fibrosis may progress to severe forms of the disease, such as liver cirrhosis (CH) and hepato-cellular carcinoma (HCC). Globally, ~ 35 million people are infected with HIV out of which 20-30% individuals are co-infected with HCV [1]. The prevalence of co-infection is higher in some key populations especially people who inject drugs due to the shared mode of transmission [2]. Although significant achievements have been made in reducing HIV/AIDS-related mortality and morbidity through successful implementation of cART, HCV-related liver disease remains a major therapeutic challenge to those co-infected with this virus. One of the major impact of HCV coinfection is the persistent low CD4^+^ T cell counts in HIV/HCV co-infected compared to HIV mono-infection individuals [3]. Conversely, co-infection of HIV adversely affects the natural history of HCV infection by multiple ways including: rapid virus replication, accelerated fibrosis and poor response to antiretroviral therapy [3]. Although, cellular immune responses elicited against HCV spontaneously clear the virus in more than 30% of infected individuals [4,5] but the majority of them fail to do so and end in chronicity.

HCV is a hepatotropic RNA virus that causes hepatitis, CH and HCC [6]. Given that HCV-specific CD8 + T cells are critical for virus control, non-specific immune response by innate effector NK cells, constituting around 30% of intrahepatic lymphocytes (IHL), too contribute to virus persistence and liver pathology [7]. However, in HIV/HCV co-infection scenario, the systemic immune dysfunction and CD4+ T cell depletion associated with HIV, remains the major factor in HCV persistence and chronic liver inflammation. Growing body of evidence have supported accelerated liver fibrosis and organ failure in HIV/HCV co-infected compared to HCV mono-infected individuals [8] especially in those with CD4 T cell count below 200 cells or at advanced stage of the HIV disease [9]. Although the cART regimen significantly restores CD4^+^ T cells in HIV mono-infection but the data are conflicting for HIV/HCV co-infection. One study reported CD4^+^ T cell recovery following 4-years of HAART [10] while other does not [9,11]. Further, HCV co-infection has been shown to negatively impact CD4^+^ T cell reconstitution following HAART [12]. Therefore, these studies suggest high mortality rate among the HCV-coinfected individuals as a consequence of severe liver disease, rather than AIDS-related illness.

CCL2, also known as monocyte chemo-attractant protein-1 (MCP-1), is a small molecular weight protein of C-C chemokine family with strong chemotactic behaviour toward monocytes, NK cells and CD4+ T cells [13,14]. Many cell types including monocytes, dendritic cells, endothelial cells (EC) and epithelial cells produce CCL2 in response to a variety of microbial insults and pro-inflammatory stimuli. Apart from leukocyte recruiting properties, role in immune homeostasis and human diseases such as cancer, infection and autoimmunity is well appreciated [15-18]. The data from ours and others laboratories strongly suggest CCL2 a supporter of HIV replication and disease progression through multiple ways (see section CCL2 supports HIV replication and disease progression). However contribution to hepatitis virus triggered chronic liver inflammation and progression to fibrosis, has recently been described both in humans and murine models of hepatitis [19-21]. CCL2 and its receptor display a varied expression and are closely linked with liver disease. For example, in non-alcoholic steatohepatitis, both CCL2 and CCR2 levels are up-regulated, causing macrophage infilteration resulting in that eventually leads to inflammation, fibrosis, steatosis and accumulation in adipose tissues [22].

Fibrosis is a key event associated with liver injury triggered by virus and other inflammatory agents. It is characterized by excessive deposition of extra-cellular matrix (ECM) components including collagens, fibronectin and proteoglycan into Desse and reduced levels of tissue inhibitor of metalloproteinase (TIMP-1), an ECM removing matrix metalloproteinase (MMP) [23]. Human liver constitutes a complex cellular environment comprised of hepatocytes, hepatic stellate cells (HSC), macrophage (Kupffer cell) and T cell subsets. HSC has been considered as the major contributor of liver fibrosis by producing inflammatory mediators and substrates required for fibrogenesis [24,25]. In this regard, HCV infected hepatoma cell derived supernatant has been shown to trigger production of most potent pro-fibrotic molecule TGF-β by HSC [26]. Notably, both HIV and HCV induce an array of inflammatory cytokines and chemokines to regulate pathogenesis of relevant diseases. Some of these include, cytokine TNF-α, TGF-β Interferons (IFNs) and ROS, and chemokine CCL2 (MCP-1), CCL3 (MIP1-α), CCL4 (MIP-1β), CCL5 (RANTES), CXCL8 (IL-8), CXCL9 (MIG), CXCL10 (IP-10) and CXCL11 (I-TAC) [22,27]. To discuss the contribution of individual molecule in hepatic fibrosis is beyond the scope of this review thus herein, we focus on one of the most relevant mediator CCL2. However a comprehensive analysis of chemokine-chemokine receptor in liver disease is described elsewhere [22].

In addition to liver-resident macrophage (Kupffer cell), human hepatic macrophages can be divided into ‘classical’ CD14^++^ CD16^−^ and ‘non-classical’ CD14^+^ CD16^+^ subsets. The later frequency found to be preferentially higher in liver fibrosis and HIV infection [28,29]. The circulating monocytes originated from the bone marrow are recruited into the liver in response to chemokines produced either by virus insult or injury. That later differentiate into functional liver macrophages as demonstrated in bone marrow and liver transplant mice models [30]. Functionally Kupffer cells are competent to sense the danger signals via TLR receptors and trigger release of inflammatory mediators including CCL2. On other hand, infiltrating monocyte-derived macrophages are found to be mostly linked to fibrosis and chronic inflammation. In this regard, experimental mouse model of fibrosis has demonstrated influx of inflammatory Ly-6C^hi^ monocytes into the liver in response to CCL2 [31] and that letter differentiate to Gr1^+^ inflammatory subset, an equevalent of human CD14^+^CD16^+^ cells.

### HIV/HCV co-infection immuno-pathogenesis

HCV-induced liver injury and subsequent progression to fibrosis is an immuno-pathological event governed by complex virus- host interactions [3,32]. Though HCV-specific infiltrating T cells are thought to be the major contributor of liver injury but in HIV co-infection setting, it is less likely that these functionally impaired cells can accelerate liver pathology. Accumulating body of evidence suggest hepatocytes and other liver cells can be infected and may serve as HIV reservoir [33-36], thus can play a regulatory role in shaping the liver specific immune responses. Interestingly, HIV and HCV can interact and reciprocally affect the natural history of each other. The potential mechanisms of HCV impact on HIV disease progression is not fully understood. One study reported activation of HIV-long-terminal repeat (LTR) promoter by HCV in transfected hepatocytes [37], while others observed increased risk of mortality among HIV/HCV co-infected individuals despite successful cART administration [9,11] as discussed in introduction part. On other hand, HIV can infect and/or activate liver cells via co-receptor CCR5 and CXCR4 to accelerate HCV-induced hepatic fibrosis [36]. In addition to virus, HIV purified proteins such as TAT [38], gp160 [39] and accessory vpu [40] can contribute to hepatic fibrosis by induction of pro-fibrotic cytokine TGF-β. Further HIV TAT has also been shown to enhance HCV replication via chemokine CXCL-10 [41]. However, depletion of Kupffer’s cell (KC) by HIV has been shown to profoundly affect the liver disease progression in HIV/HCV co-infected individuals [42]. Taken together above studies suggest the reciprocal effects and consequences of viral interactions on the liver injury.

Immuno-pathology of HIV/HCV co-infection associated liver disease is multi-factorial [43]. Several hypothesis of HIV-mediated acceleration of liver injury have been described in HIV/HCV co-infection [44,45]. This includes HIV-associated immune dysfunction, defective antiviral CD8+ T cells responses and reduced CD4/CD8 ratio. The perturbed cellular ratio may contribute to liver damage, as higher proportion of CD8+ T cells are suggested relatively more fibrogenic than CD4+ T cells [46]. Another important event that may contribute to liver fibrosis is the direct activation of HSC by HIV gp120 or via pro-inflammatory mediators induced in response to infection [45,47]. Moreover, the potential mechanisms by which both the pathogens can contribute to liver damage may include, (1) HIV and HCV-triggered production of reactive oxygen species (ROS) that signals via c-Jun N-terminal kinase (JNK), extracellular signal-related kinase (ERK) and p38 mitogen activated protein kinase (p38MAPK) through NFkB to up-regulate TGF-β production and decrease of matrix metaloprotease-3 (MMP3) [48,49]. (2) Stimulation of HCV-infected hepatocytes by HIV gp120 to induce HCV replication via TGF-β [49]. (3) Induction of hepatocyte apoptosis by HIV and HCV to trigger pro-fibrotic activity of HSC as observed in both HIV/HBV and HIV/HCV co-infections [50-53].

### Impact of HIV-associated microbial translocation on liver fibrosis

**Figure 1:**
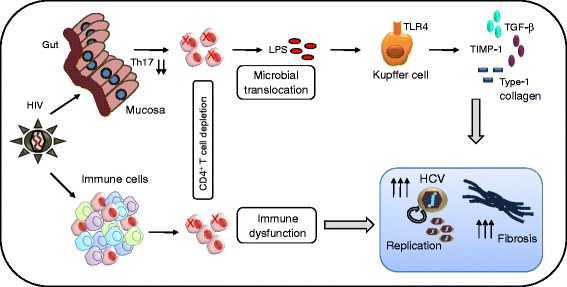
**Adverse effects of HIV on HCV replication and liver fibrosis.** HIV-associated systemic immune dysfunction and microbial translocation are the critical factors that drive HCV replication and fibrosis in HIV/HCV co-infected individuals. Depletion of CD4^+^T cells in conjunction with Th17 cells causes microbial translocation that dispenses their products in the blood circulation. Activation of Kupffer cells via TLR4 produces numerous fibrotic mediators including TGF-β, TIMP-1 and type-1 collagen. Taken together both systemic immune dysfunction and microbial translocation contribute to enhanced HCV replication and liver fibrosis.

Immune activation is a hallmark of advanced HIV disease associated with chronic T cells activation and sustained plasma levels of pro-inflammatory cytokines. Systemic immune dysfunction and mucosal CD4+ T lymphocytes depletion from lymphoid tissues of gastrointestinal (GI) tract of HIV positive individuals are thought to be the major driving force behind this event. The mucosal permeability and subsequent microbial translocation leads to production of high levels of soluble(s) CD14, sCD163 and IL-6 by activated macrophages in response to microbial product, lipopolysaccharide (LPS) [54]. In addition to HIV, this phenomenon has also been observed in HIV/HCV and HIV/HBV co-infection, where a higher levels of plasma LPS, sCD14 were detected in coinfected than mono-infected individuals [54-56]. As liver cells are constantly exposed to gut-derived LPS via portal circulation, there is a high likelihood of KC and HSC activation to produce inflammatory mediators such as TNF-α, TGF-β and CCL2 [25,57]. Under such situation, KC has been attributed with larger role since binding of LPS to their TLR4 releases large amount of pro-fibrotic molecule TGF-β, TIMP-1 and collagen type 1 [58] to set the stage for initiation of fibrosis (Figure 1).

The association of microbial translocation and contribution of LPS to liver inflammation in HIV/HCV co-infection setting has recently been recently appreciated [59-61]. These studies suggest LPS a key component account for rapid disease progression and organ failure. In addition to microbial products, a high quantity of innate anti-viral cytokine IFN-α and associated T cell activation, immensely contribute to virus replication and CD4+ T cell death in HIV/HCV co-infected than HIV mono-infected individuals [62,63]. Given the occurrence of microbial translocation in both HIV-mono and HIV/HCV co-infections, strategies should be developed to reduce overt immune activation in order to contain progressive liver diseases.

### CCL2 supports HIV replication and disease progression

Host chemokines induced by HIV may either support or inhibit virus replication. For example, C-C chemokine CCL3, CCL4, CCL5 and C-X-C chemokine CXCL12 (SDF-1α) has been described as potent inhibitors of R5 and R4 HIV strains by binding to their respective co-receptors CCR5 and CXCR4 [64-66]. Thus, early production of the above chemokines in lymph nodes can be beneficial in containing the virus spread. In this context, researcher have succeed in developing of CCR5 analogue, AOP-RANTES and antagonist Maraviroc [67,68], the latter has now progressed to clinical practice. Unlike HIV inhibitory C-C family members, CCL2 is thought to support HIV replication by multiple ways [69-72] indicating a functional dichotomy within C-C chemokine family members.

**Figure 2:**
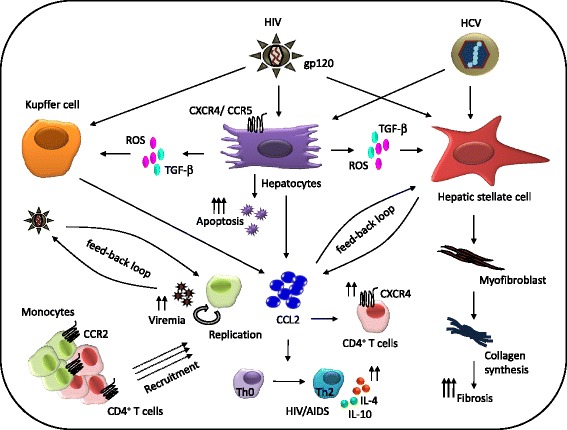
**CCL2-mediated synergistic effects of HIV/HCV co-infection on liver fibrosis.** Proposed model explains migration of HIV into the liver to infect or activate Kupffer cells, hepatocytes and HSCs directly or indirectly via ROS and TGF-β, to create CCL2 rich inflammatory milieu. CCL2 may act on HSC in a positive feed-back loop manner to accelerate fibrosis via myofibroblast and collagen synthesis. Elevated CCL2 in the liver could also recruit HIV permissive CD4^+^ T cells and monocytes into the liver for next round of virus replication in a positive feed-back loop manner. This leads to persistence of a high HIV viremia. In addition, elevated CCL2 can polarize helper T cells (Th0) cells into IL-4 and IL-10 secreting Th2 phenotype, a strong determinant HIV disease progression. Further, CCL2 can induce CD4^+^ T cells to express higher levels of CXCR4, an HIV co-receptor. Overall this scheme depicts how a complex cellular interactions and virus cross-talks via CCL2 can potentially drive hepatic fibrosis HIV replication in HIV/HCV co-infection scenario.

A number of clinical data including ours have shown elevated CCL2 in the serum [70,71] and cerebrospinal fluid (CSF) [73] of HIV subjects that significantly correlates with plasma viral load. Further, we reported a differential CCL2 expression by HIV-infected viremic and aviremic individuals, suggesting active virus replication leads to greater CCL2 induction and thus diseases severity compared to suppressed one [70,71]. The potential mechanisms of CCL2 mediated enhanced HIV pathogenesis has been described elsewhere [72,74]. This includes, (1) the positive feed-back loop model where recruitment of HIV permissive CCR2+ monocytes/macrophages and CD4+ T cells at the site of infection for new round of replication [70,75]. (2) induction of co-receptor CXCR4 on resting CD4+ T cells [76]. (3) up-regulation of CXCR4 through IL-4 [77] (4) differentiation of helper T cell (Th0) cells to type-2 helper T cells (Th2) [78], a hallmark of HIV/AIDS. (5) enhancement of HIV virion release [79]. A summary of above mechanisms in conjunction with HCV-coinfection is described later in the article (Figure 2).

Evidence of CCL2 production is also observed in non-human primate (NHP) model of simian human immunodeficiency virus (SHIV) infection [80], supporting the notion that CCL2 is indeed an important and relevant factor in HIV infection. Further, considering one of the most common HIV/Mtb co-infection scenario, CCL2 is believed to exacerbate the disease pathogenesis by adversely affecting the natural history of both the pathogens [81]. Although CCL2 is mostly produced by monocytes in the peripheral blood, the inflammatory CD14^+^CD16^+^ monocyte subset has been found to be the major source of CCL2 in HIV-infected individuals [29] as well as in liver fibrosis [28].

### CCL2 accelerates HCV-induced liver fibrosis

A series of inflammatory mediators are up-regulated during HCV infection and associated liver diseases such as CH and HCC [82-84]. Some of these mediators are, reactive oxygen species (ROS), CCL2, IL-6, CXCL8 and CXCL10 released by activated KC and hepatocytes [46]. In addition to these, HCV induced early interferons (IFNs) production by KC triggers production of CCL2. High levels of CCL2 may recruit CCR2^+^ leukocytes including; monocytes, NK cells, CD4^+^ T cells into the liver to start inflammatory reactions [85,86]. Compelling clinical evidence suggest an increased CCL2 and its receptor CCR2 mRNA transcripts in HCV infected liver specimens [87,88] that positively correlates with disease severity [89]. The relevance of CCL2 in liver diseases was further supported by studies on liver transplantation [90], HCC [91] and fibrosis regression with pharmacological inhibitor of CCL2 in mice [21]. Thus, above clinical studies and experimental models clearly suggest CCL2 a key mediator of hepatic fibrosis and their potential blocking may offer improved clinical outcome.

A detailed association between chemokines and liver diseases has been described elsewhere [22]. Some of the important chemokines and their cognate receptors relevant to HCV and fibrosis include, CCL2 and receptor CCR2 on monocyte/macrophages and HSC; CCL3, CCL4, CCL5 and their receptor CCR1 and CCR5 on natural killer (NK), CD8 and Th1 cells; CXCL9, CXCL10 CXCL11 and receptor CXCR3 on NK, CD8, Th1 and HSC. Further example chemokine mediated liver injury includes, persistence of elevated serum CXCL-10 and CXCL8 levels in the recipients following liver transplantation, who developed higher hepatic necro-inflammation and fibrosis [92]. Beside regulating leukocytes trafficking, CCL2 possess strong angiogenic characteristics as an inducer of VEGF expression [46] shown by infiltrating macrophages in mouse model of inflammatory associated progressive fibrosis [20]. Further, sustained levels of CCL2 has been considered critical in both triggering liver injury and subsequent development of fibrosis and thus, can serve as predictor of progression towards cirrhosis [19]. It is the cross-talk between HSC and HCV-infected hepatocytes that derive immune-pathogenesis and onset of fibrosis [93] potentially via an autocrine CCL2 loop [94-97]. Apart from HCV infection of hepatocytes, viral core protein NS5A has been found to increase ROS via mitochondrial insult [98]. ROS activate KC to release CCL2 that transforms HSC to into pro-fibrogenic myofibroblast to secrete α-smooth muscle actin and fibriller collagens I and III (Figure 2). Moreover, host genetic make-up of HCV-infected individuals can greatly influence the outcome of the liver diseases. For example, CCL2 polymorphism is found to be associated with significantly higher hepatic expression specifically in those individuals with advance liver fibrosis [99].

### Mechanisms of CCL2-mediated HIV:HCV interactions in liver fibrosis

The natural infection of both HIV and HCV is affected by their presence in the same host. Compared to HCV, the effect of HIV on HCV infection is considered more deleterious (described in mechanisms of HIV/HCV immuno-pathogenesis section). Given that CCL2, an important factor associated with pathogenesis of both the viruses, it is important to understand the impact on liver cellular microenvironment and virus-triggered hepatic fibrosis. We herein summarize the potential mechanisms (Figure 2) that includes, (1) both HCV and HIV can induce release of CCL2 by KC, hepatocyte and HSC, either directly or indirectly via TGF-β and ROS produced by hepatocytes [48,49]. (2) This up-regulates CCL2 levels in the liver to recruit that CCR2^+^ monocytes and CD4^+^ T cells from the hepatic portal blood to the site of infection in a feed-back loop manner, resulting in rapid HIV replication and subsequent increase in viremia. (3) CCL2 can also act on HSC in a positive feed-back loop [36] to transform activated HSC into myofibroblast and collagen synthesis. In addition to their role in liver fibrosis, it is argued that high levels of CCL2 can severely affect the course of HIV infection by up-regulating HIV-co-receptor CXCR4 on CD4^+^ T cells [76] and polarization of helper T cells towards Th2 phenotype [78], a hallmark of progressive HIV disease. In summary there is complex virus-virus and host-virus interaction in HIV/HCV co-infection, and CCL2 is certainly an important factor that mediates these above cross-talks.

### Therapeutic management of HIV/HCV co-infection

Significant reduction in HIV associated mortality and morbidly has been achieved with the advent of cART. However, co-infection of HCV has posed major clinical challenge as proportion of HIV/HCV co-infected individuals develop CH and HCC. Generally the treatment of HCV mono-infection involves 24–48 weeks of antivirals consisting pegylated interferon-alpha (pegIFN-α) plus ribavirin (RBV) [9]. A regimen that also found to be effective in cases of HIV/HCV-coinfections, achieving a sustained virological response (SVR) ranging between 25-50% with cure rate of 70-80% for HCV geneotype 2 and 3 and relatively reduced SVR of 18-38% for the genotype 1 and 4 infections [100]. Although, pegIFN/RBV therapy has significantly reduced the liver associated co-morbidities in co-infected individuals, the adverse effect on central nervous system (CNS) and hepatic toxicity has led to a decline in treatment uptake as reported in EuroSIDA cohort studies [101]. Therapeutic treatment of HCV/HIV was tremendously boosted with the advent of multiple new direct acting antivirals (DAA) including HCV protease and polymerase inhibitors, and HCV NS3-4A inhibitor telaprevir and boceprevir in 2011 [100] and the data from pilot studies demonstrated significantly improved the outcome compared to standard pegIFN/RBV alone [102,103]. However, the drug is not in use anymore potentially due to drug-drug interaction between inhibitors of HIV and HCV, frequent adverse effect such toxicity of liver, CNS, high cost and pill burdens. Now, the European Association for the Study of the Liver (EASL) recommends a new IFN-free therapy against HIV/HCV coinfection that includes sofosbuvir plus daclatasvir/simprevir/ledipasvir. The newly developed AbbVie 3D regimen, consists of combination of HCV NS3/4A protease inhibitor ABT-450 with ritonavir, the NS5A inhibitor ombitasvir (ABT-267), and the NS5B RNA polymerase inhibitor dasabuvir (ABT-333) with or without ribavirin have shown high cure rate in phase III clinical trials, and expected to be in clinical practice very soon.

In addition to antivirals, chemokine and chemokine receptor based blockers and antagonists could provide an attaractive immuno-therapeutic approach against HIV/HCV co-infection associated liver diseases. This startegy has proven success in HIV infection, where CCR5 antagonist, Maraviroc effectively suppress HIV replication in infected individuals [68]. Given the immune-therapeutic potentials of CCL2 blockade against breast and prostate cancer [15,16], the CCL2-CCR2 target based approach may prove beneficial in reducing HIV/HCV co-morbidities. In this regard, CCR2 antagonist, CCX140 and inhibitor BMS-741672, has already been tested for Phase II studies of liver fibrosis. While, NOX-E36, a CCL2 blocker [21] and the dual CCR2/CCR5 blocker, Ceniceriviroc (Tobira Therapeutics Inc, San Francisco, CA) have shown appreciating results in rodent models of fibrosis. Given that some of the above tests in animals found to be successful, the human test is yet to prove their efficacy. Therefore, more efforts needed to understand the immune-pathological events to employ above approach to treat infection associated liver injury.

## Conclusion

The majority of the literature supports the notion that mortality among HIV/HCV co-infected individuals occurs due to liver failure rather than AIDS-related complications. Since fibrosis is an immune-pathological event it would wise to target those factors which are induced by both the viruses and are critical in liver diseases severity. CCL2 is one such pro-inflammatory molecule that could be targeted for anti-inflammatory strategies since CCL2 blockade strategy has already been implemented clinically in breast and prostate cancer immune-therapy [15,16]. Further understanding of intra-hepatic inflammatory network, microbial translocation and metabolic factors hold the promise for developing new therapeutic approaches. Perhaps humanized animal models supporting HIV and HCV infections can provide more mechanistic and therapeutic answers to this problem.
